# Supplementary material for: Polarization sensing of network health and seismic activity over a live terrestrial fiber-optic cable
Source: Commun Eng. 2024 Jul 4;3:91. doi: 10.1038/s44172-024-00237-w (PMC11224415; doi:10.1038/s44172-024-00237-w)
Supplement: Supplementary file 2 — Supplementary Information [file 44172_2024_237_MOESM2_ESM.pdf]

Supplementary Information for "Polarization sensing of network health and seismic activity over a live terrestrial fiber-optic cable"

Charles J. Carver*[1] and Xia Zhou[1]

[1]Columbia University, 500 W 120th St, New York, USA
*Corresponding author, cjc@cs.columbia.edu

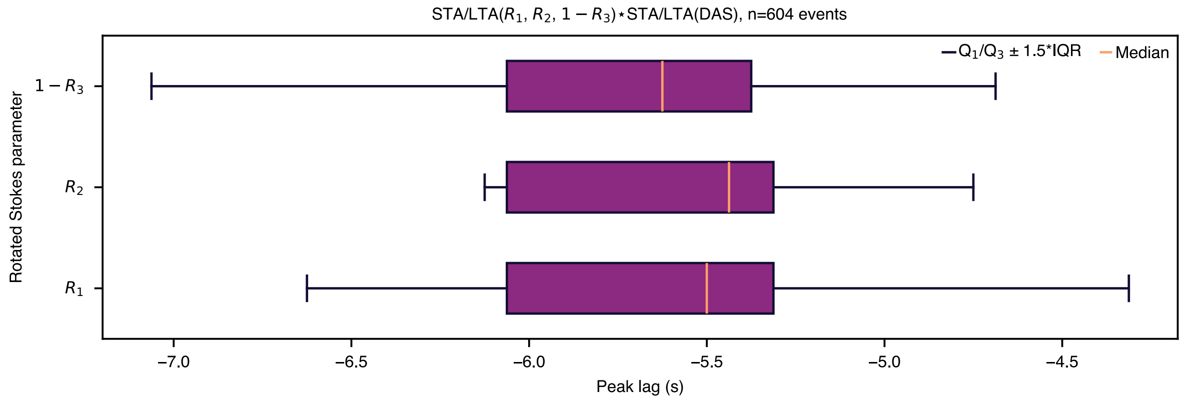

Supplementary Figure 1: Distribution of cross correlation peak lags between n=604 state-of-polarization events and distributed acoustic sensing events. Each box extends from the 1st to the 3rd quartile, with caps indicating the maximum/minimum lag falling within 1.5 the interquartile (IQR) range. The orange lines indicate the median peak lag for each rotated Stokes parameter used in the cross-correlation, i.e., -5.5 s for R_1, -5.4 s for R_2, and -5.6 s for $1 - R_3$. The average median lag across all Stokes parameters is -5.5 s, with the negative sign indicating that Stokes perturbations were detected before distributed acoustic sensing perturbations.

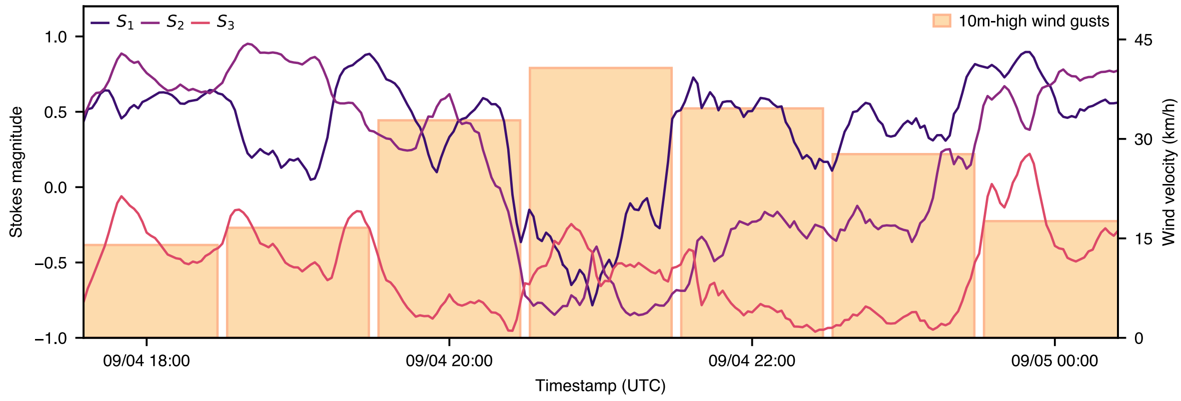

Supplementary Figure 2: High-intensity wind gusts correlated with state-of-polarization fluctuations. The wind gusts, measured 10 m in the air, shake the aerial portions of the optical fiber and generate similar Stokes perturbations to those caused by seismic surface wavefronts. This strengthens our hypothesis that the seismic wavefront has a nonnegligible impact on aerial sections of the fiber.
